# Dimerization of cAMP phosphodiesterase-4 (PDE4) in living cells requires interfaces located in both the UCR1 and catalytic unit domains

**DOI:** 10.1016/j.cellsig.2014.12.009

**Published:** 2015-04

**Authors:** Graeme B. Bolger, Allan J. Dunlop, Dong Meng, Jon P. Day, Enno Klussmann, George S. Baillie, David R. Adams, Miles D. Houslay

**Affiliations:** a Institute of Pharmaceutical Sciences, King's College London, London SE1 9NH, United Kingdom; b Departments of Medicine and Pharmacology, University of Alabama, Birmingham, AL 35294, USA; c Institute of Chemical Sciences, Heriot-Watt University, Riccarton, Edinburgh EH14 4AS, Scotland, United Kingdom; d Institute of Cardiovascular and Medical Science, College of Medical, Veterinary and Life Sciences, University of Glasgow, Glasgow G12 8QQ, Scotland, United Kingdom; e Max Delbrueck Center for Molecular Medicine, German Centre for Cardiovascular Research (DZHK), Berlin, Germany

**Keywords:** PDE4, Phosphodiesterase, cAMP, cyclic AMP, Dimerization, Dimerisation, Rolipram

## Abstract

PDE4 family cAMP phosphodiesterases play a pivotal role in determining compartmentalised cAMP signalling through targeted cAMP breakdown. Expressing the widely found PDE4D5 isoform, as both bait and prey in a yeast 2-hybrid system, we demonstrated interaction consistent with the notion that long PDE4 isoforms form dimers. Four potential dimerization sites were uncovered using a scanning peptide array approach, where a recombinant purified PDE4D5 fusion protein was used to probe a 25-mer library of overlapping peptides covering the entire PDE4D5 sequence. Key residues involved in PDE4D5 dimerization were defined using a site-directed mutagenesis programme directed by an alanine scanning peptide array approach. Critical residues stabilising PDE4D5 dimerization were defined within the regulatory UCR1 region found in long, but not short, PDE4 isoforms, namely the Arg^173^, Asn^174^ and Asn^175^ (DD1) cluster. Disruption of the DD1 cluster was not sufficient, in itself, to destabilise PDE4D5 homodimers. Instead, disruption of an additional interface, located on the PDE4 catalytic unit, was also required to convert PDE4D5 into a monomeric form. This second dimerization site on the conserved PDE4 catalytic unit is dependent upon a critical ion pair interaction. This involves Asp^463^ and Arg^499^ in PDE4D5, which interact in a *trans* fashion involving the two PDE4D5 molecules participating in the homodimer. PDE4 long isoforms adopt a dimeric state in living cells that is underpinned by two key contributory interactions, one involving the UCR modules and one involving an interface on the core catalytic domain. We propose that short forms do not adopt a dimeric configuration because, in the absence of the UCR1 module, residual engagement of the remaining core catalytic domain interface provides insufficient free energy to drive dimerization. The functioning of PDE4 long and short forms is thus poised to be inherently distinct due to this difference in quaternary structure.

## Highlights

• In a yeast 2-hybrid system we show that long PDE4 isoforms dimerize.
• Scanning peptide array and mutagenesis located two dimerization surfaces.
• One surface maps to the regulatory UCR1 region found only in long forms.
• A second locates to the core catalytic domain.
• PDE4 long and short forms differ in quaternary structure.

## 1 Introduction

Cyclic AMP is a ubiquitous second messenger that plays a pivotal role in regulating many key cellular processes [1–5]. cAMP signalling in mammalian cells is compartmentalised so that spatially distinct sub-populations of the cAMP effectors, PKA and Epac can differentially regulate a range of distinct intracellular processes [1,5–7]. The differential activation of such effectors is achieved through gradients of cAMP formed by spatially distinct sub-populations of both adenylyl cyclase and cAMP degrading phosphodiesterases [1,8]. 9 different PDE sub-families are capable of degrading cAMP and, in addition to exhibiting cell-type specific patterns of expression, they are differentially located in cells, conferring distinct roles upon enzymes from this super-family [2,3,9–13]. Differences in intracellular targeting, coupled with regulated changes in both their activity and targeting elicited by post-translational modification, place PDEs firmly as critical enzymes regulating cellular function [1]. Indeed, the ability to generate inhibitors selective for each PDE sub-family has been judiciously exploited in order to both generate therapeutic agents and garner understanding of the functional significance of these enzymes [3,9,14].

Members of the PDE4 enzyme family play a pivotal role in cell functioning. These enzymes are encoded by four genes (*PDE4A, PDE4B, PDE4C, PDE4D*), which generate over 20 distinct isoforms through alternate mRNA splicing and the use of distinct promoters [1,2,9,10,15–18]. PDE4 isoforms critically define the compartmentalization of cAMP signalling through their ability to be recruited to specific signalling complexes, where they shape cAMP gradients in a temporal and spatial manner [1]. As such, individual isoforms have specific, non-redundant roles acting in defined intracellular compartments; as elucidated through dominant negative, siRNA-mediated knockdown and peptide displacement approaches [19–22]. Their functioning in these distinct locales is dynamically regulated through phosphorylation by kinases such as PKA [23–26], Erk [27–29], MK2 [30,31] and AMPK [30] as well as modification by ubiquitination [32] and sumoylation [33].

Many proteins can undergo dimerization, which can lead to functional differences [34]. In this regard, enzymes from the various PDE families have a highly conserved catalytic unit and many sub-families are also characterized by distinct, paired domains located N-terminal to this. These include the Ca^2 +^/calmodulin binding domains of PDE1, the Gaf domains of PDE2, PDE5, PDE6, PDE10, PDE11 and the UCR1/2 domains of PDE4 [2,9,15,17]. Such domains have been implicated in dimer formation [35–46].

Alternative mRNA splicing of all four PDE4 genes yields a plethora of isoforms. These can be sub-categorised as ‘long’ forms that possess both UCR1 and UCR2 regulatory domains, ‘short’ forms that lack UCR1 and ‘super-short’ forms that lack UCR1 and have a truncated UCR2 [1,3,9,10,14–16,18,47]. Also identified are ‘dead-short’ forms that lack both UCR1 and UCR2 and have a truncated catalytic unit, making them catalytically inactive [48].

There is now good evidence that PDE4 isoforms can form dimers [41–43]. These elegant studies have shown that dimerization in cells is restricted to the long, but not the short, isoforms as UCR1 is fundamental to this process. Consistent with this, 2-hybrid studies using isolated domains have demonstrated that UCR1 can interact with UCR2 and this might, through *trans* interaction, facilitate assembly of a long isoform dimer [49]. Notwithstanding the apparent requirement for UCR1, X-ray crystallographic analyses of active, but highly truncated PDE4 core catalytic units, reveal that the isolated catalytic domain can dimerize, at least under crystallisation conditions, despite the absence of UCR1 and UCR2 [50]. The dimerization interface within the catalytic unit comprises a focal contact surface at a C2 symmetry axis that is bounded at each end by an Asp/Lys charge pairing that is conserved in all four PDE4 sub-families [50].

Here we use two novel approaches to gain further insight into the nature of PDE4 dimers formed in living cells, focusing on the widely expressed PDE4D5 long isoform as an exemplar [51]. Amongst other things, this isoform has particular functional importance in regulating the β_2_-adrenoceptor through its ability to bind to the β-arrestin signalling scaffold [52–56], and also in the migration and polarity of cells through its ability to bind to the RACK1 signalling scaffold [21,32,53,56,57]. In one approach, which we describe here, we employed a yeast 2-hybrid methodology to evaluate dimerization in living cells and, in a second approach we used scanning peptide array analyses to determine the details of the PDE4 dimerization site located in the long form-specific UCR1 domain. These studies have allowed us to engineer a catalytically active mutant form of PDE4D5 that, unlike the native dimeric enzyme, exists as a monomer in living cells.

## 2 Materials and methods

### 2.1 Materials

Primary antibodies used are rabbit-polyclonal anti-VSV (Abcam Ltd., Cambridge, CB4 0FL, UK), mouse polyclonal anti-HA (Covance, Alnwick, NE66 2JH, UK), mouse anti-FLAG-horseradish peroxidase conjugate and VSV (vesicular stomatitis virus)-affinity agarose beads were from Sigma (Gillingham, Dorset, SP8 4XT, UK). Anti-GST antibody (Santa Cruz/Insight Biotechnology Ltd, Wembley, Middlesex HA9 7YN, UK). Secondary antibodies used are anti-mouse horseradish peroxidase linked Ig (GE Healthcare, Amersham Place, Little Chalfont Bucks, HP7 9NA, UK) and anti-rabbit horseradish peroxidase linked Ig (Sigma, Gillingham, Dorset, SP8 4XT, UK). Stock solutions of rolipram were prepared in DMSO. Bradford reagent was from Bio-Rad (Hemel Hempstead, Herts, HP2 4PD, UK). Polyfect transfection reagent was from Qiagen (Lloyd Street North, Manchester M15 6SH). Protease inhibitor tablets were from Roche. Plasmid DNA was prepared using the QIAprep® Spin Miniprep kit from Qiagen (Lloyd Street North, Manchester M15 6SH). [8-^3^H[cAMP was from GE Healthcare (Amersham Place, Little Chalfont Bucks, HP7 9NA, UK) and unlabelled cAMP together with all other biochemicals were from Sigma (Gillingham, Dorset, SP8 4XT, UK). NuPAGE was from Invitrogen (Paisley PA4 9RF, UK). ECL reagents were from Pierce/ThermoFisher (Northumberland, NE23 1WA, UK).

### 2.2 Yeast 2-hybrid analyses

Yeast 2-hybrid techniques are identical to those used previously by us to identify and analyse protein–protein interactions [57,58]. In all experiments, one of the two interacting proteins was expressed as “bait”, as a *LexA* DNA-binding domain fusion. The second protein was expressed as “prey”, as a *GAL4* activation-domain protein. In some experiments, a third protein (*i.e.*, either RACK1 or β-arrestin2) was also expressed, but as a native species and not as a fusion protein. All bait were expressed either in pLEXAN or in pBridge as *LexA* DNA-binding domain fusions. All prey were expressed in pGADN as *GAL4* activation-domain fusions. All non-fusion co-expressed proteins were expressed in pBridge (*i.e.*, pBridge expresses two proteins, one as a *LexA* DNA-binding domain fusion and the other with only a nuclear localization signal). All proteins were targeted to the nucleus. Human isoforms of all proteins were used in these experiments. In all figures, standards were added (the oncoproteins RAS and RAF1). All interactions were evaluated in the *Saccharomyces cerevisiae* strain, L40 with our standard filter β-galactosidase assay. Except where indicated otherwise, all experiments shown here have been repeated at least twice, with identical results. In the experiments in Fig. 6e, a quantitative β-galactosidase assay was used, as we have described previously [49].

All mutations in PDE4D5 were generated by the circular mutagenesis method, using Pfu polymerase (Agilent Technologies) and were verified by sequencing prior to use.

### 2.3 SPOT synthesis of peptides and peptide array probing

Peptide libraries were generated by automatic SPOT synthesis on Whatman 50 cellulose membranes using Fmoc (9-fluorenylmethyloxycarbonyl) chemistry with the Autospot-Robot ASS 222 (Intavis Bioanalytical Instruments, 50933 Koeln, Germany). The interaction of peptide spots with GST and GST-fused purified proteins by overlaying the cellulose membranes with 10 μg/ml of recombinant protein was determined as described previously by us in some detail [32,52,53,59–62]. Bound recombinant proteins were detected with specific primary antisera and complementary HRP-coupled secondary antibody as for immunoblotting.

### 2.4 Cell culture and transfection

HEK (human embryonic kidney)-293 cells were maintained in DMEM (Dulbecco's modified Eagle's medium) supplemented with 2 mM glutamine, 10% (v/v) foetal bovine serum (Sigma), 1% penicillin/streptomycin (100 units/ml) in an atmosphere of 5% CO_2_ and 37 °C. All cells were transiently transfected with PolyFect.

### 2.5 Immunoprecipitation and Western blotting

Cells were transiently transfected with wild-type PDE4D5-VSV, PDE4D5-FLAG, PDE4D5-HA and the indicated mutant forms. At 48 h post-transfection, cells were lysed using a 3T3 lysis buffer containing 20 mM HEPES (pH 7.4), 50 mM NaF, 10% (v/v) glycerol, 1% (w/v) Triton X-100, 10 mM EGTA, 30 mM sodium pyrophosphate and protease inhibitor cocktail. Cell lysates were pre-cleared with Protein G beads and then used for immunoprecipitation of VSV-tagged proteins. Cell lysates were incubated with VSV-affinity agarose beads for 2 h at 4 °C. After centrifugation at 18,000 g for 3 min, beads were washed three times with lysis buffer. Laemmli [63] sample buffer was used to elute the immunoprecipitated VSV-fusion proteins and the resulting material was resolved by NuPAGE. Proteins were transferred on to nitrocellulose membranes and subjected to Western blotting with detection by enhanced chemiluminescence (ECL).

### 2.6 PDE activity assay

PDE activity was measured using radioactive cAMP hydrolysis assay that has been described previously in detail [64,65].

## 3 Results

### 3.1 PDE4D5 forms a dimer in living cells

**Fig. 1:**
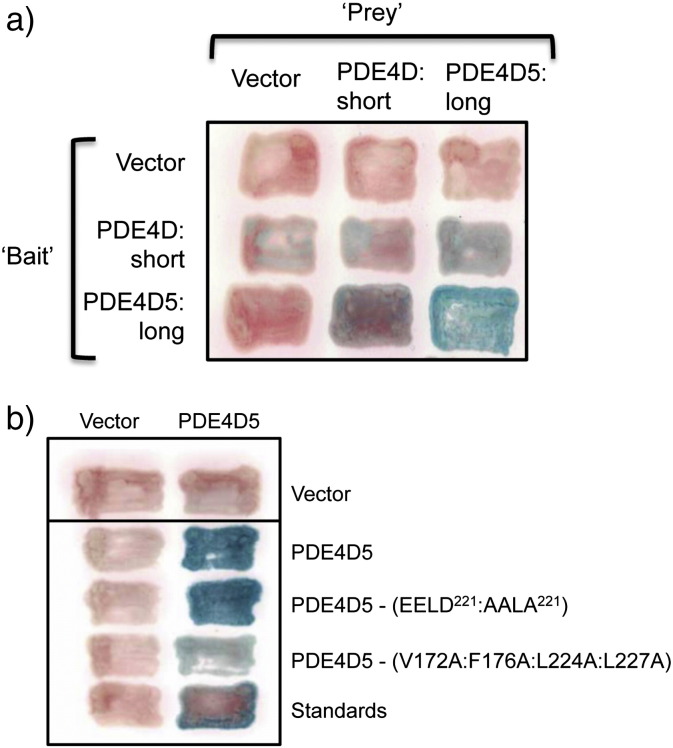
PDE4 dimerizes in living cells. Yeast 2-hybrid experiments using PDE4D5 as both ‘bait’ and ‘prey’. PDE4D5 was expressed unmutated (“wild-type”) or with the indicated point mutations. PDE4D5 was expressed unmutated (“wild-type”) or with the indicated deletion or point mutations. All patches in each column use identical ‘prey’ and all patches in each row use identical ‘bait’, as described in Materials and methods. Controls are vectors alone and standards are Ras^V12^–Raf, as done before by us [57]. Positive interactions, assessed with a filter β-galactosidase assay, produce blue patches, while negative interactions produce pink patches. (a) Full-length PDE4D5 (amino acids 1 to 749; “long”), or PDE4D5 lacking its unique N-terminal domain and UCR1, but containing UCR2 and the catalytic region (amino acids 206 to 747 of PDE4D5; “short”), was expressed as either a fusion to LexA (rows) or to GAL4 (columns) and the various mutants tested for interaction. (b) The EELD:AALA mutant reflects that ablating the UCR1:UCR2 interaction [49] while the VFLL:AAAA mutation reflects that used to ablate PDE4D3 dimerization [42]. This shows data typical of experiments performed at least 3 times.

Using both immunoprecipitation and gel filtration approaches with PDE4D2 (short) and PDE4D3 (long) as exemplars, it has been proposed that long, but not short, PDE4 isoforms adopt a dimeric state when isolated from cell extracts [41–43]. Consistent with such a notion, when we expressed the long PDE4D5 isoform in yeast, as both bait and prey in a 2-hybrid assay, it is clearly evident that PDE4D5 monomers interact to form dimers in living cells, yielding blue, β-galactosidase positive, patches (Fig. 1a, b).

It has been shown that the UCR1 domain, which is lacking in short forms, is critical to the formation of dimers [41–43]. While we show here that the ‘long’ PDE4D5 isoform can interact strongly with itself in living cells to form dimers, as exemplified here using yeast two-hybrid assays (*i.e.*, can dimerize; Fig. 1a), in agreement with previous studies of others [41–43], a construct that we engineered for this study to approximate a short form, critically by the removal of the UCR1 of PDE4D5, exhibited a profoundly attenuated ability to dimerize in two-hybrid assays (Fig. 1a). Thus, as previously stated by others [41–43], the dimerization of PDE4 long forms requires UCR1 while PDE4 short forms, which lack UCR1, fail to dimerize significantly.

We have previously demonstrated that UCR1 and UCR2 interact to form a functional module [49]. Critical to this are 3 negatively charged residues in UCR2 that are located within a contiguous cluster (218-EELD-221; PDE4D5 numbering), which bind to a positive patch within the largely hydrophobic UCR1 and whose mutation, to alanine, ablates UCR1:UCR2 interaction [49]. Mutating these residues, all to alanine, in PDE4D5 (EELD:AALA), however, failed to ablate PDE4D5 dimerization, as assessed in 2-hybrid assays in the present studies (Fig. 1b).

Richter and Conti [42] employed a helical wheel analysis in order to direct a mutagenesis approach to gain insight into residues important in determining UCR1:UCR2-based long form dimerization. Their M2 mutation, made in PDE4D3, targeted the conversion of a VFLL set of non-contiguous residues (PDE4D3 numbering is V100, F104, L152, L155; PDE4D5 numbering is V172, F176, L224, L227) to alanine, which ablated dimer formation in their hands [42]. Here we investigated the cognate mutant in PDE4D5 and, consistent with their discovery, we showed that it did indeed compromise dimerization of this long form in a living cell assay (Fig. 1b).

We have found that in most if not all instances where PDE4 is involved in binding to a partner protein then more than one binding surface is invariably involved, which contributes to binding fidelity [53,54,59–61]. Thus, as PDE4D5 dimerization was not fully ablated by mutation of either of the contiguous EELD or non-contiguous VFLL clusters (Fig. 1b), we decided to investigate, using peptide array technology, whether other regions of PDE4D5 were involved in stabilising its dimeric state.

### 3.2 Probing PDE4 long form interaction interfaces using scanning peptide arrays

**Fig. 2:**
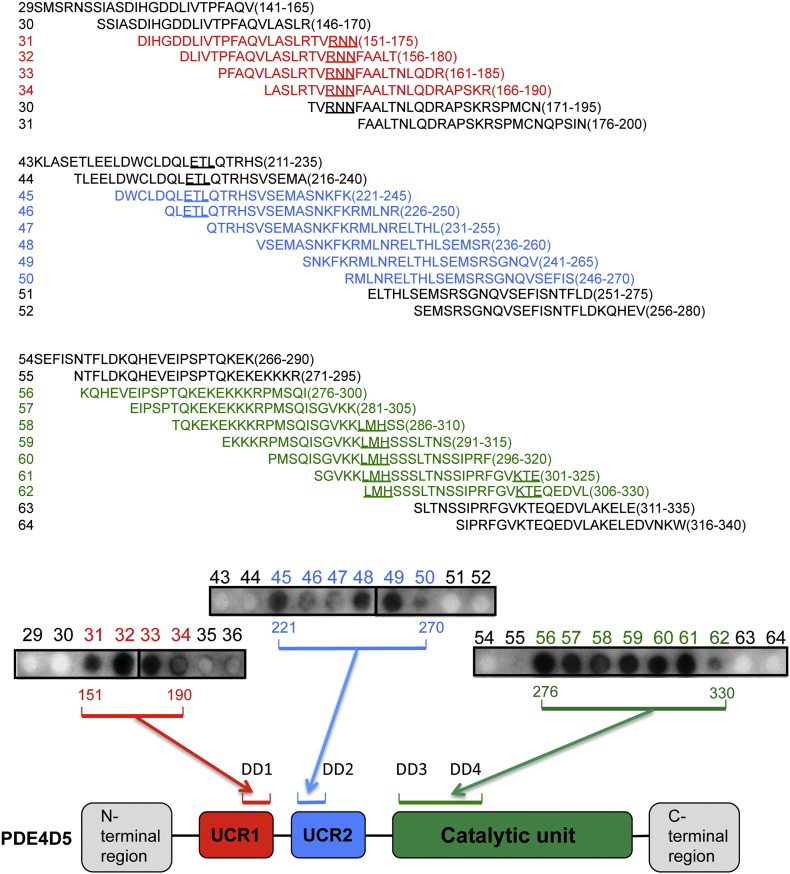
Delineation of PDE4D5 dimerization sites by peptide array. An immobilized library of 25-mer peptides sequentially shifted by 5 amino acids along the entire sequence of PDE4D5 was probed with either purified PDE4D5-GST or GST alone. Dark spots represent areas of interaction between PDE4D5-GST and the PDE4D5 peptide array. Areas of interaction are identified by a colour code where red represents UCR1, blue represents UCR2 and green represents sequences within the catalytic unit. No interaction was detected between GST alone and the PDE4D5 peptide array (data not shown).

In order to uncover further interactions required for oligomeric self-association of PDE4D5 we used peptide array analysis, which provides a novel and powerful technology for gaining insight into the basis of specific protein–protein interactions [32,52,53,59–62]. In order to do this a library of overlapping peptides (25-mers), each shifted by 5 amino acids across the entire 745 amino acid sequence of PDE4D5 was spot-synthesised on cellulose membranes. This immobilized peptide library was subsequently probed with a purified, recombinant GST-PDE4D5 fusion protein whose binding to protein spots was assessed, immunologically, with positive interactions identified as dark spots. Undertaking such an analysis we identified positive interactions within not only UCR1 but also the UCR2 and catalytic regions (Fig. 2).

**Fig. 3:**
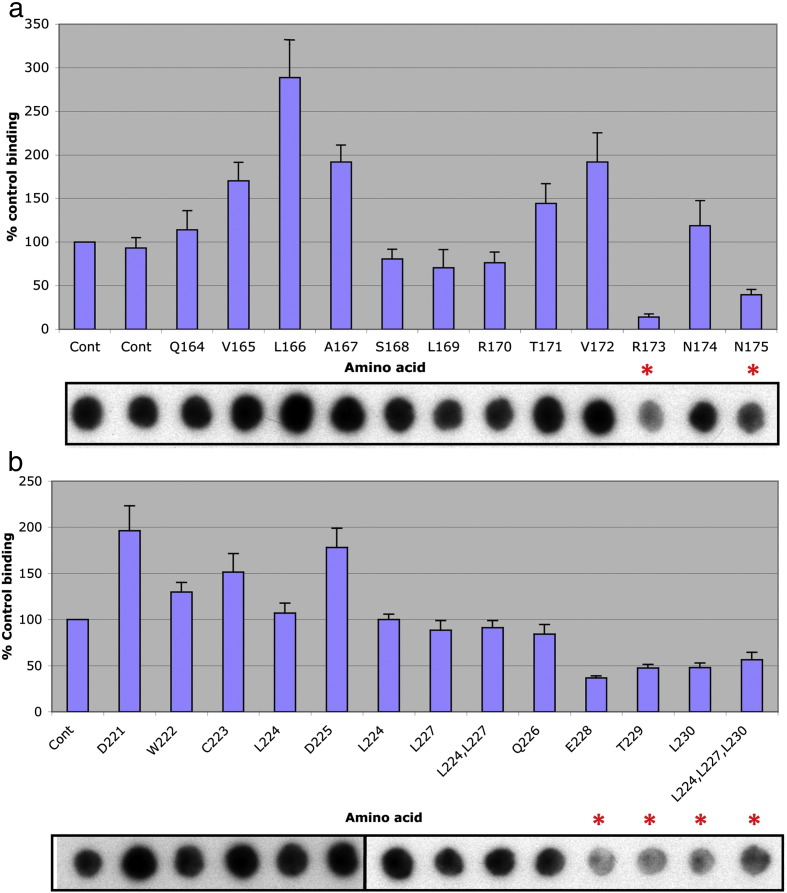

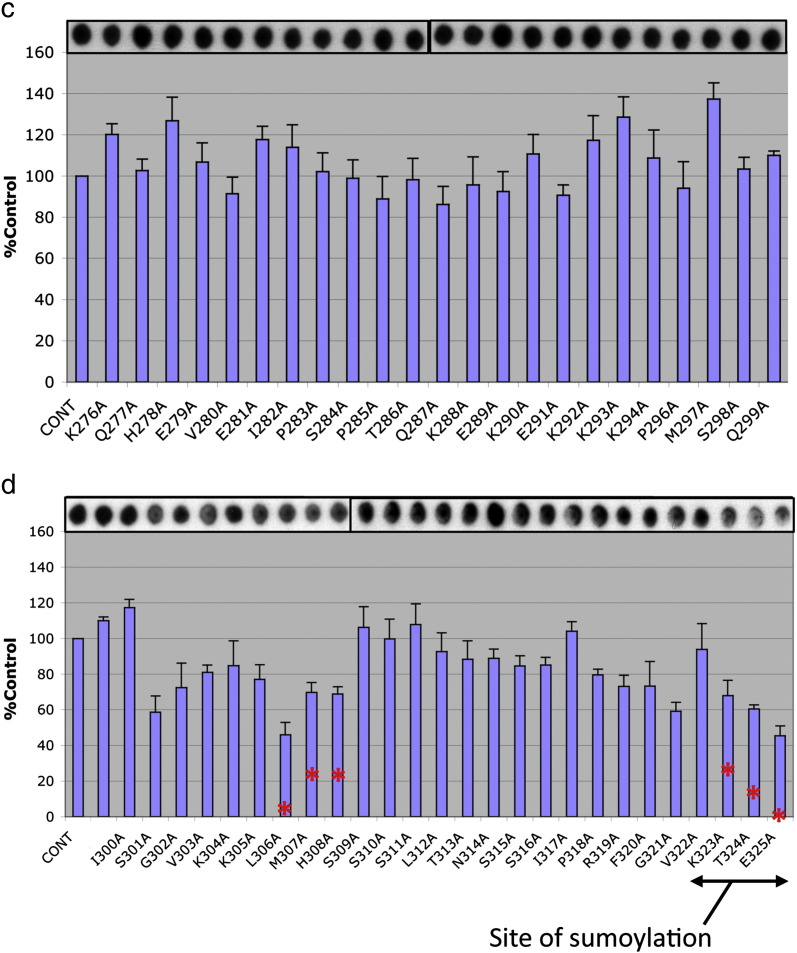
Alanine scanning pinpoints amino acids that are important for PDE4D5 dimerization. Alanine scanning analysis (where each of the indicated residues is replaced with alanine or aspartate if residue is an alanine) was undertaken on three PDE4D5 self-association domains highlighted in Fig. 2. Arrays were overlayed with PDE4D5-GST or GST alone. Dark spots represent areas of interaction between PDE4D5-GST and the PDE4D5 peptide array. Cont = control spot chosen from data depicted in Fig. 2 and where all 25 amino acids correspond to the native sequence. Data represents the mean plus S.E. from three independent experiments. No interaction was detected between GST alone and the PDE4D5 peptide array (data not shown).

To define further the specific amino acids involved in PDE4D5 binding to this peptide array, we screened a family of peptides derived from distinct 25-mer parent peptides that positively interacted with PDE4D5. The peptide progeny from specific 25-mer parents were generated such that each had a single substitution, to alanine, of successive amino acids in the sequence to form a scanning peptide array that was probed with recombinant GST-PDE4D5 fusion protein. Sections of these arrays are shown here to demonstrate those amino acids whose substitution to alanine engendered a significant and reproducible compromised interaction with GST-PDE4D5 (Fig. 3; highlighted by a red asterisk). This identified R^173^ and N^175^ within UCR1; E228, T229 and L230 in UCR2 and L306, M307, H308 and K323, T324, E325 within the catalytic unit (Fig. 3).

**Fig. 4:**
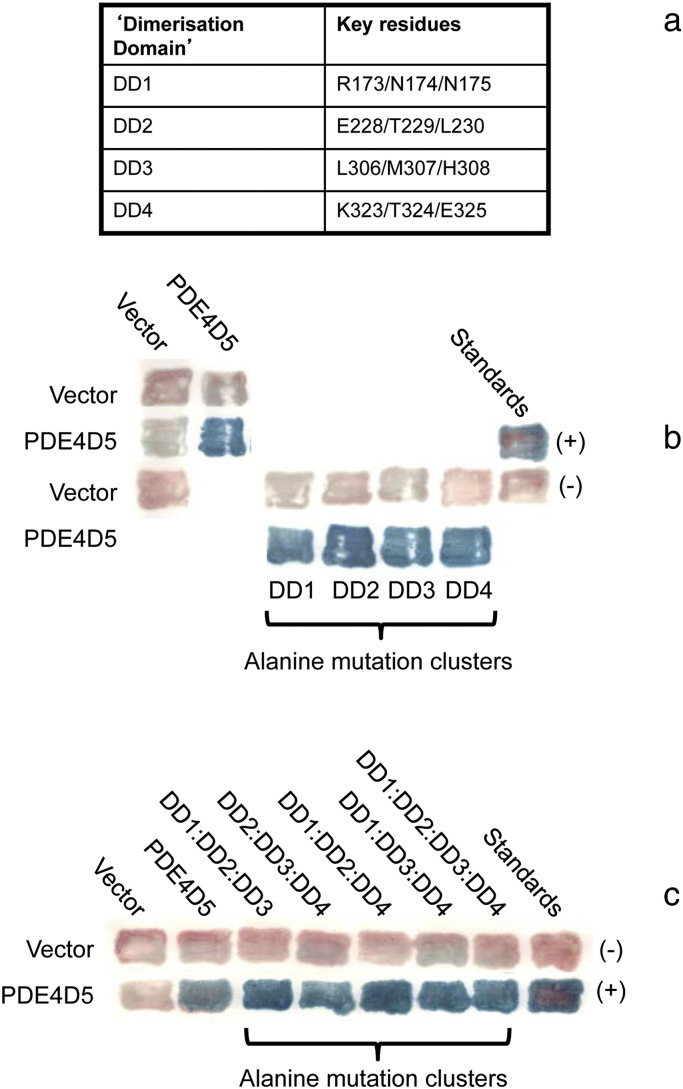
Characterization of ‘Dimerization Domain’ (DD) mutants. (a) Amino acid co-ordinates for the 4 DD regions on the PDE4D5 protein used throughout this study. (b) Mutations in individual DD domains do not ablate the interaction. The amino acids in each individual DD domain were mutated to alanine and tested for their effect on PDE4D5 dimerization in 2-hybrid assays, as in Fig. 1. (c) Mutations in combinations of DD domains do not ablate the interaction. Combinations of DD domain mutants were tested for their effect on PDE4D5 dimerization. This shows data typical of experiments performed at least 3 times.

We then set out to determine whether these were involved in PDE4D5 homodimerization, evaluating four regions that we termed DD1 (R173, N174 and N175), DD2 (E228, T229 and L230), DD3 (L306, M307, H308) and DD4 (E228, T229 and L230) (Fig. 4a). Firstly we mutated, to alanine, each set of residues within these clusters and assessed the regions individually as to whether they compromised dimerization, which they appeared not to do (Fig. 4b). Then we assessed combinations of 3 of these clusters and, finally, all four DD clusters (in the quad-PDE4D5 construct), when mutated to alanine in order to determine whether they compromised dimerization, which they appeared not to do (Fig. 4c).

### 3.3 Demonstrating that the ion pair stabilising the dimeric surface evident in crystallographic studies of the core PDE4D catalytic unit is required for PDE4D5 dimerization in living cells

**Fig. 5:**
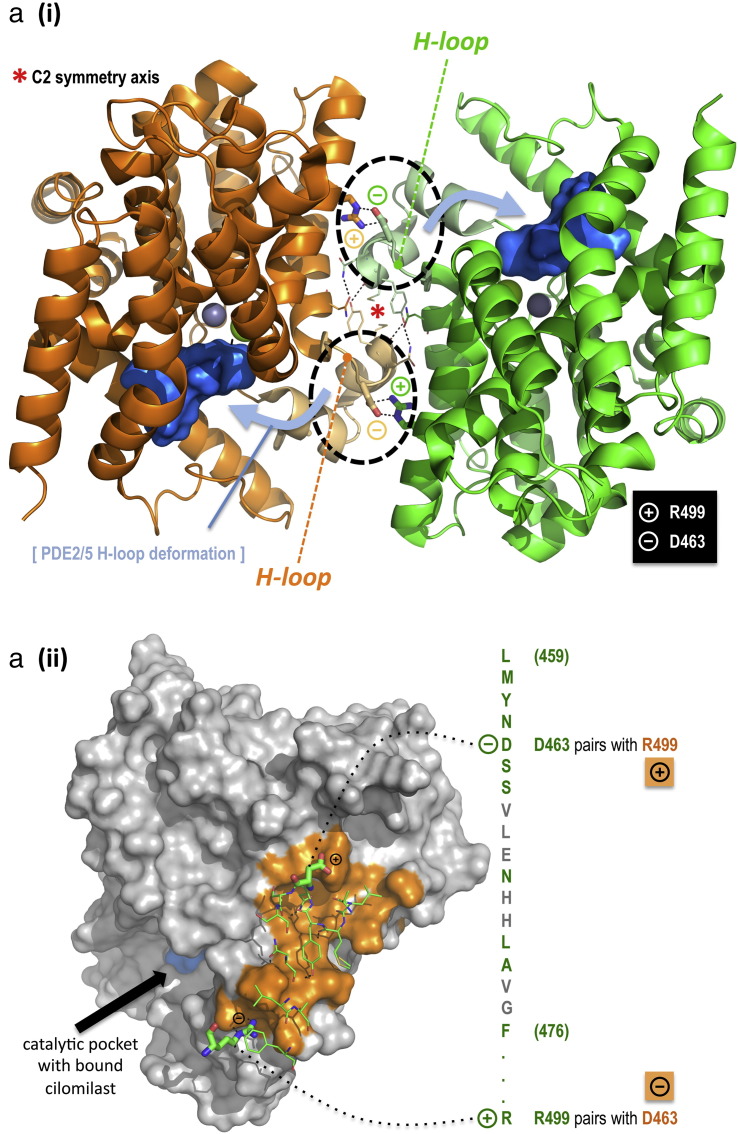

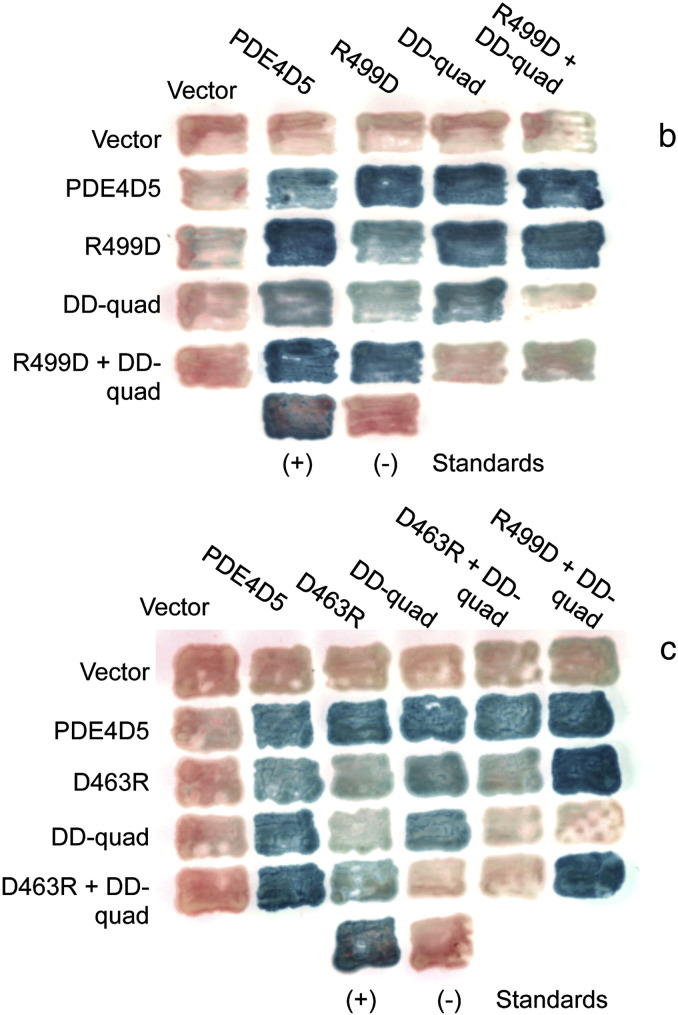
Structure of dimeric PDE4 core catalytic domain assembly and the effect of the R449D mutation on dimerization. (a) (i) The dimeric PDE4D core catalytic domain with bound cilomilast inhibitor (blue surface) is shown (PDB: 1XOM; [73]). Assembly is centred on a C_2_ symmetry axis (asterisk) at the junction of helices-9 and -10; residues contributing to the focal interaction are highly organised through a network of hydrogen bonds. Helix-11 makes a single point of contact in the dimer through R499 in a salt bridge to H-loop residue, D463. Key residues contributing to the dimerization interface are only partially conserved in other PDE families. In contrast to the highly organised H-loop structure in PDE4, the corresponding region (dotted circle) in PDE2 and PDE5 is conformationally mobile and dynamically folds into the catalytic pocket (blue arrow) to regulate catalytic activity (see Discussion). (ii) Surface rendition derived from (i) highlighting the core catalytic domain dimerization interface on one subunit (orange) and contact residues from the second subunit (green stick). (b) Mutations in PDE4D5 were tested for their ability to ablate dimerization: The ‘DD-quad’ mutant is a combination of all four DD domain mutations where each are described individually in Fig. 4a. The R449D-PDE4D5 mutant and the combined R499D + DD-quad-PDE4D5 mutant were also studied. Two-hybrid assays were used as in Fig. 1. The data show that neither the R499D-PDE4D5 mutant nor the DD-quad-PDE4D5 mutant ablated the interaction, but that the combined R499D + DD-quad-PDE4D5 mutant markedly ablated the interaction. (c) Rescue of the charge effect of the R499D-PDE4D5 mutant by the D463R-PDE4D5 mutant. The D463R-PDE4D5 mutant was tested singly and in combination with the DD-quad-PDE4D5 mutant. The data show that the D463R-PDE4D5 mutant effectively rescued the ablation of the interaction seen with the combined R499D + DD-quad-PDE4D5 mutant. This shows data typical of experiments performed at least 3 times.

While aggregation of full length PDE4 isoforms has militated against structural studies of such physiologically relevant species, there is a wealth of 3-D structural data on truncated species that represents the highly conserved core catalytic unit found in each of the four PDE4 sub-families (see *e.g.* [66]). Interestingly, these studies reveal that the ‘naked’ PDE4 catalytic units crystallise from solution as homo-dimers. Detailed examination of the dimerization interface showed that it comprises a highly organised focal network of hydrogen bonded interactions and hydrophobic contacts augmented by a critical Asp–Arg ion pairing [50], which in PDE4D5 involves Asp463 and Arg499 (Fig. 5a). While these two charged residues appear to play no role in maintaining the structural integrity of an individual catalytic unit, mutation of either Asp:Arg or Arg:Asp ablated the ability of a PDE4D core catalytic construct to dimerize [50]. Interestingly, this site was not discovered in our peptide array analyses, which at least in part likely arises from a failure of short 25-mer peptides to conserve the very precise 3-D conformation of the PDE4 catalytic subunit in this tightly folded region.

We thus set out to investigate any potential role of this ion pair in the dimerization of full-length PDE4D5 (Fig. 5b). The R449D mutation failed to ablate dimerization when this mutant was expressed either alone or with native PDE4D5 or quad-PDE4D5 (where all four DD sites identified by peptide array analyses were mutated to alanine; see Fig. 4a for details) and, finally, a R499D-quad-PDE4D5 construct (Fig. 5b). However, in marked contrast to either the R499D-PDE4D5 or quad-PDE4D5 construct, which underwent homo-dimerization, we discovered that incorporating both sets of disruptions in the R499D-quad-PDE4D5 construct then it singularly failed to undergo dimerization (Fig. 5b). Furthermore, the R499D-quad-PDE4D5 mutant failed to interact with the quad-PDE4D5 mutant, although it did interact with the R499D-PDE4D5 mutant (Fig. 5b). It would thus appear that to ablate dimerization of PDE4D5 effectively in living cells then disruption of the ion pair stabilised catalytic unit dimerization interface uncovered in crystallography studies is needed together with disruption of key regions located N-terminal to the catalytic unit.

It could be argued that disruption of the ion pair formed by Asp463 and Arg499 in the PDE4D5 catalytic unit might indirectly compromise dimerization by undermining the overall structural integrity of the domain. Against such a contention, crystallography studies show that these residues do not maintain the tertiary structure of the catalytic unit [50]. Nevertheless we set out to address this question directly through charge reversal rescue experiments. If indeed maintenance of the catalytic domain tertiary structure is independent of the residue identity at positions 463 and 499, then it should be possible to establish a reversed D499-R463 salt bridge to reinstate dimerization with the R499D-quad-PDE4D5 mutant. Pleasingly our experiments confirmed this (Fig. 5c). Thus, whereas the D463R-quad-PDE4D5 and R499D-quad-PDE4D5 mutants failed to homodimerize when individually expressed, dimerization capacity was indeed rescued when the two constructs were jointly expressed, through establishment of a complementary heterodimeric D463-D499:R463-R499 pairing at the catalytic domain interface.

We also noted that neither the R499D-quad-PDE4D5 nor the D463R-quad-PDE4D5 mutants interacted with the DD-quad-PDE4D5 mutant but both were capable of interacting with wild-type PDE4D5 (Fig. 5c). An interesting difference however, was that while R499D-quad-PDE4D5 interacted equally as well with wild-type-PDE4D5 and the single R499D mutant (Fig. 5b), D463R-quad-PDE4D5 showed compromised binding to the single D463R-PDE4D5 mutant compared to wild-type-PDE4D5 (Fig. 5c). Interestingly, the single mutations of each of these residues forming the catalytic dimer-stabilising ion pair behaved similarly in that each single mutant showed, compared to wt-PDE4D5, reduced binding when expressed individually or paired with quad-PDE4D5 (Fig. 5b, c). These studies show that single mutations in this ion pair have dramatic and specific consequences for the PDE4D5 dimerization process, which clearly requires an additional site located N-terminal to the catalytic unit.

### 3.4 The DD1 motif in UCR1 is critical for PDE4D5 dimerization

**Fig. 6:**
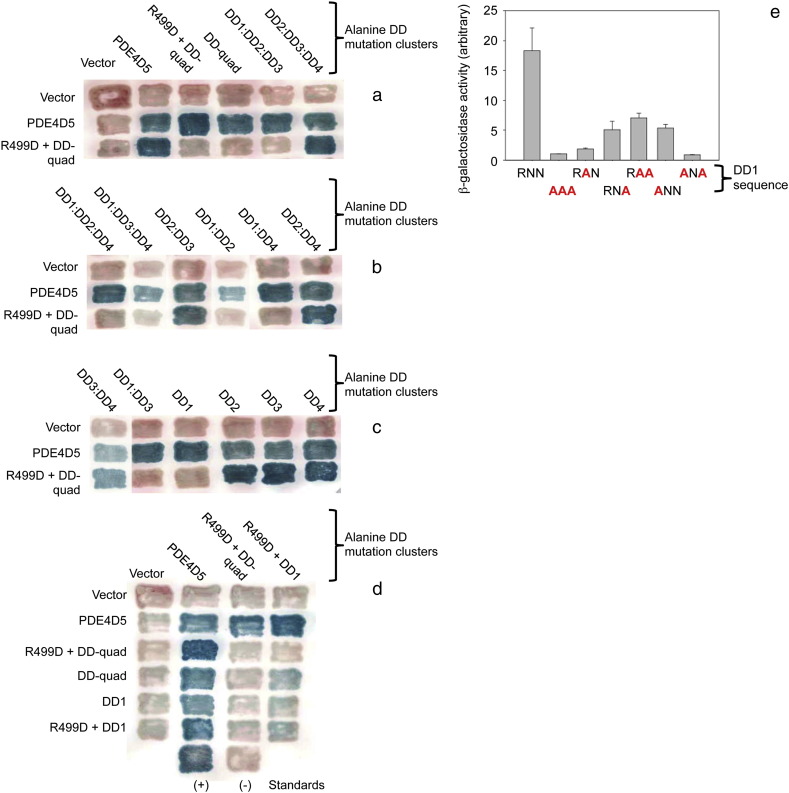
Key effects of DD1 region mutants on dimerization. (a,b,c) The effects of various combinations of DD domain mutants were studied in filter β-galactosidase assays for their effect on their interaction with R499D + DD-quad-PDE4D5 mutant. Two-hybrid assays were used as in Fig. 1. The data show that combinations containing mutations in DD1 ablated the interaction, while mutations in other combinations of DD domains had minimal effect. (d) Comparison of the DD1-PDE4D5 mutants in *cis* and *trans* with the R499D-PDE4D5 mutant. The R499D, DD-quad, and DD1 PDE4D5 mutants when singly and in combination, were tested against the same array of mutants. The data show that the DD1-PDE4D5 mutant blocks the interaction when present in either half of the dimer (*i.e.*, regardless of whether it is present in the ‘prey’ or ‘bait’ construct.) (e) Quantitative analysis of DD1 region individual amino acid mutants: Individual amino acids in PDE4D5-DD1 (sequence RNN, Fig. 4a) were mutated to alanine and tested in a quantitative β-galactosidase assay for their effect on their interaction with R499D + DD-quad-PDE4D5. The data show that restoration of one or two amino acids to wild-type increases the interaction, at least in some cases, but never back to wild-type. This suggests either that (1) All three amino acids in the RNN motif contribute directly to the interaction; or that (2) Even single amino acid mutations in this area cause sufficient localized distortion of the PDE4D5 structure that they attenuate the interaction. This shows data typical of experiments performed at least 3 times.

We next set out to determine whether all of the DD regions identified in our peptide array analyses are critical for PDE4D5 homo-dimerization using constructs where the indicated combinations of DD1-4 amino acids were mutated to alanine (see details of residues in Fig. 4a). Probing with the R499D-quad-PDE4D5 construct we showed that while it failed to interact with itself (R499D-quad-PDE4D5) and also failed to interact with DD-quad-PDE4D5 and DD1:DD2:DD3-PDE4D5, it was able to interact with DD2:DD3:DD4-PDE4D5 (Fig. 6a), suggesting that of these regions identified by peptide array then the UCR1-derived, DD1 (Fig. 2) contributes key residues to the pertinent region. Consistent with the importance of DD1, we showed that while R499D-quad-PDE4D5 failed to interact with either DD1:DD3:DD4-PDE4D5, DD1:DD2-PDE4D5 or DD1:DD3-PDE4D5, it did interact with DD2:DD3-PDE4D5, DD2:DD4-PDE4D5 and DD3:DD4-PDE4D5 (Fig. 6b, c). Finally, R499D-quad-PDE4D5 failed to interact with the single DD1 cluster mutation of DD1-PDE4D5, while it interacted with all of the single DD2-PDE4D5, DD3-PDE4D5 and DD4-PDE4D5 cluster alanine mutations (Fig. 6c).

On this basis we probed the DD1-PDE4D5 mutation further with other mutant PDE4D5 species. These showed (Fig. 6d) that while DD1-PDE4D5 interacted with wild-type-PDE4D5 it not only failed to interact with R499D-quad-PDE4D5 but also failed to interact with R499D-DD1-PDE4D5, showing that the minimal mutation of R499D and DD1 is all that is required to ablate PDE4D5 dimerization in living cells.

To gain further insight into the importance of the three amino acid residues mutated in DD1 (Fig. 4a) we assessed them through either individual or paired alanine mutations in 2-hybrid experiments involving paired R499D-*X*-PDE4D5 constructs, where ‘X’ was either R173A or N174A or N175A (Fig. 6e). This clearly showed that alanine mutation of any of these three residues severely compromised PDE4D5 dimerization, with the effects of R173A and N174A being most profound (Fig. 6e).

### 3.5 PDE4D5 dimerization and its disruption in mammalian cells

**Fig. 7:**
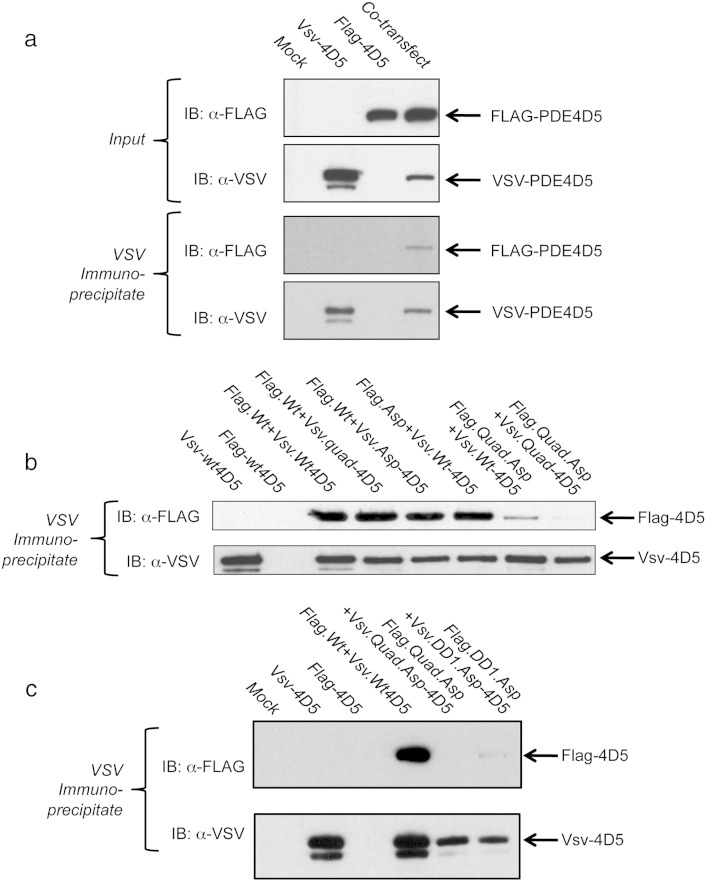
PDE4D5 dimerization in mammalian cells. The indicated Flag and vsv-tagged PDE4D5 wild-type and mutant constructs were co-expressed in HEK293 cells. Cells were then disrupted and lysates taken for either immunoblotting with both anti-Flag and anti-vsv antisera or immunoprecipitated with anti-vsv antisera prior to immunoblotting with both anti-Flag and anti-vsv antisera. The data shown are representative of experiments performed at least three times using different transfected cell preparations.

Having demonstrated PDE4D5 dimerization for the first time in living cells, we set out to determine whether the R499D-quad-PDE4D5 and R499D-DD1-PDE4D5 species were monomeric when expressed in mammalian cells (Fig. 7). Richter and Conti have previously determined [41,42] that when the PDE4D3 long isoform is transiently expressed in mammalian cells, as two differently tagged species, then immunoprecipitation using antibodies to one tag co-immunoprecipitated both tagged forms of PDE4D3, consistent with its dimerization. We show here that in HEK293 cells transiently expressing both flag- and vsv-tagged PDE4D5 long isoform constructs, then immunoprecipitation with one antisera co-immunoprecipitates both tagged forms of PDE4D5 (Fig. 7a). Such studies demonstrate the dimerization of PDE4D5, which is consistent with the studies and conclusions of Richter and Conti [41,42] using the PDE4D3 long isoform.

Such co-immunoprecipitation is ablated when the flag-Quad-R499D-PDE4D5 construct is paired with vsv-Quad-PDE4D5 and severely compromised when either the flag-Quad-R499D-PDE4D5 construct is paired with vsv-WT-PDE4D5 (Fig. 7b) or flag-DD1-R499D-PDE4D5 construct is paired with vsv-DD1-R499D-PDE4D5 (Fig. 7c). Such data are consistent with the 2-hybrid studies showing that dimeric PDE4D5 can be forced into a monomeric state by DD1 and R499D mutation.

**Fig. 8:**
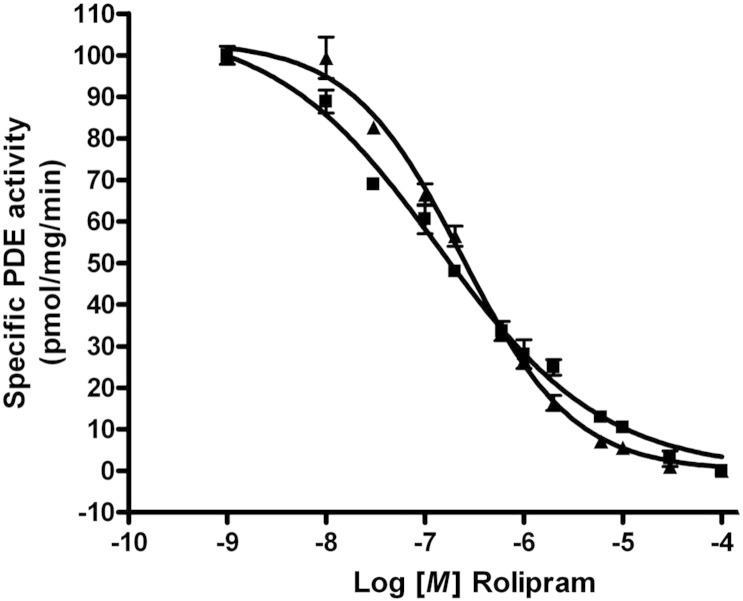
Dimeric and monomeric PDE4D5 are similarly inhibited by rolipram. Lysates of HEK293 cells transfected to express either wild-type- (dimeric) or DD1-R499D- (monomeric) PDE4D5 were used to assess the ability of increasing concentrations of rolipram to inhibit their activity using 1 μM cAMP as substrate. Data shows means ± SD for a single experiment with triplicate assays. Data from replicates of such experiments was used to determine IC_50_ values of 1.4 ± 0.2 × 10^− 7^ M for dimeric wild-type PDE4D5 and 2.3 ± 0.3 × 10^− 7^ M for monomeric DD1-R499D-PDE4D5, with only four amino acids mutated (SD, n = 3 separate experiments).

The conversion of PDE4D5 into a monomeric species by these discrete mutations has little effect on the efficacy of the pan-PDE4 inhibitor [14], rolipram to inhibit this isoform (Fig. 8). Expressed transiently in HEK 293 cells, dimeric wild-type PDE4D5 exhibited an IC_50_ value of 1.4 ± 0.2× 10^− 7^ M while monomeric DD1-R499D-PDE4D5, with only four amino acids mutated, exhibited an IC_50_ value of 2.3 ± 0.3 × 10^− 7^ M (SD, n = 3 separate determinations).

## 4 Discussion

Many proteins undergo dimerization and some even undergo oligomerization that can lead to tertiary changes in structure, which may then elicit functionally important alterations in their activity and distribution [34]. Such changes can have either physiological or pathophysiological consequences.

Cyclic AMP phosphodiesterases provide the sole route for degrading the ubiquitous second messenger, cAMP in cells [1–3,9]. As such they are poised to play critical regulatory roles. Members of the ubiquitously expressed cAMP-specific PDE4 family are of particular importance, influencing major processes such as inflammation, learning and memory, cell cycle and the cardiovascular system [14,16,18,67]. Critical to this is the ability of specific isoforms to act as nodes that respond to inputs from other signalling systems through their ability to undergo multi-site phosphorylation [10,18,30,31]. They also perform a pivotal role in underpinning compartmentalised cAMP signalling by determining targeted cAMP degradation [1,12]. This occurs as a consequence of their ability to be sequestered at specific intracellular sites/‘signalosomes’ in an isoform-specific fashion through protein–protein interactions with distinct anchoring proteins.

The four-gene PDE4 family encodes over 20 different isoforms that are defined by their unique N-terminal regions together with sub-family specific variations in sequence of the catalytic unit, C-terminal and linker regions [17]. Catalytically active species can then be categorised into either long forms, which possess the regulatory UCR1 and UCR2 regions and short forms that lack UCR1. The UCR1/UCR2 module plays a pivotal role in regulating PDE4 catalytic activity, where the UCR2 region has an auto-inhibitory role [9,16,17,47]. Regulation is mediated through multi-site phosphorylation of UCR1, where phosphorylation by PKA elicits both activation [23–26] and an ability to ablate inhibitory phosphorylation of the PDE4 catalytic unit by Erk [27,29]. UCR1 also provides the site for phosphorylation by MK2 [31], which serves to attenuate the degree of activation conferred by PKA phosphorylation and, in the case of PDE4D5, a site for mono-ubiquitination by the β-arrestin-sequestered E3 ligase, Mdm3, which gates poly-ubiquitination of the PDE4D5 isoform-specific N-terminal region [32].

A growing body of evidence, garnered from biochemical studies, indicates that PDE4 long isoforms adopt a dimeric quaternary structure and that UCR1 performs a pivotal role in underpinning this [41–43]. In contrast, PDE4 short forms appear to be exclusively monomeric. 2-hybrid and biochemical approaches akin to those described in this study have shown that UCR1 and UCR2 interact with each other [49] and, as such, this interaction has the potential to contribute to either dimer formation when binding in a *trans* configuration or to stabilise a monomer when binding in a *cis* configuration. Indeed, the charged residues suggested to stabilise UCR1:UCR2 interaction may reflect those important for stabilising a monomeric configuration where UCR1 interacts internally with UCR2 in a *cis*-configuration, as their mutation in the PDE4D3 long form failed to ablate its ability to dimerize [41] and, similarly, we confirm this here with PDE4D5 (Fig. 2). However, our scanning peptide array approach (Figs. 2, 3, 4) has allowed us to identify a critical Arg173, Asn174 and Asn175 cluster in UCR1 (PDE4D5 numbering) whose mutation, to alanine, ablates dimer formation as long as the catalytic unit dimerization site is co-disrupted by mutation of one or both of the charged species that stabilise this surface (Asp463 and Arg499 in PDE4D5). Unfortunately there is no complete structure of UCR1 and UCR2 that would allow us to define this dimerization surface and the residues that these groups might link to. Possibilities include UCR1 interacting with conserved regions on either itself or UCR2 or the catalytic unit. However, one would presume that the interaction is driven by charge–charge interaction involving Arg173 and H-bonding involving both Asn174 and Asn175. We believe that the RNN motif identified here in peptide array experiments and confirmed by mutagenesis as important in PDE4 dimerization represents a critical core unit whose mutation causes minimal overall change in the structural integrity of the enzyme. This is because RNN:AAA mutation of itself does not alter the dimerization status of PDE4D5 until it co-presents with the R:D catalytic dimer site mutation.

The VFLL:AAAA combination mutation identified previously as ablating dimerization in PDE4D3 [42], and shown here to seriously compromise it in PDE4D5 (Fig. 1), may exert its profound effect by directly destabilising a dimerization interface involving elements from UCR1 and UCR2. Thus, based on sequence analysis with a helical wheel model [42], the valine and phenylalanine from this residue set have been tentatively mapped to a contiguous hydrophobic surface in a postulated UCR1 C-terminal α-helix [UCR1(C)]; the two leucines were proposed to map to a complementary hydrophobic surface in a helically organised N-terminal half to UCR2 [UCR2(N)]. It is therefore tempting to suggest that the UCR1(C) and UCR2(N) regions may assemble as a 4-helix bundle in dimeric PDE4 long forms (*vide infra*), such that hydrophobic surfaces running along the proposed helices are a major contributory feature to a dimerization interface. While the VFLL residue set may directly contribute to such an interface within a helix bundle, none of these residues were identified as individually critical for interaction when mutated separately in our alanine scanning peptide array analysis. At present we cannot definitively state whether the combined mutation of these residues compromises dimerization because they directly contribute to a binding interface or because they indirectly perturb adjacent regions that do. In particular the valine and phenylalanine of the VFLL set immediately flank the DD1 site (172-VRNNF-176) in UCR1(C), while the two leucines immediately precede the DD2 motif (224-LDQLETL-230) that we have identified in UCR2(N).

The RNN UCR1 dimerization motif identified here in PDE4D long forms is identical in PDE4B long forms (*e.g.* Arg165, Asn166 and Asn167 cluster in PDE4B3) and highly conserved in PDE4A (*e.g.* Arg192, Ser193 and Asn194 cluster in PDE4A4) and PDE4C (*e.g.* Arg134, Ser135 and Asn136 cluster in PDE4C3) long forms where the central Asn group is replaced by Ser, which is also capable of H-bond formation. Intriguingly, in these two sub-families then the Ser group can potentially be modified by phosphorylation, which might provide a means of dynamically regulating dimerization in these two PDE4 sub-families.

A fundamental discovery uncovered by our approach is the critical importance of the catalytic unit dimerization interface, first identified from crystallographic analyses [50], to the stabilisation of PDE4 long isoforms dimerization expressed in living cells. This is stabilised by an ion pair, namely Asp463 and Arg499 in PDE4D5, binding in a *trans* configuration [50]. As with the DD1 cluster in UCR1, this ion pair is conserved in the catalytic units of all four PDE4 sub-families (see *e.g.* Asp498 in PDE4A4, Asp456 in PDE4B3; Asp421 in PDE4C3; Arg534 in PDE4A4, Arg492 in PDE4B3; Arg457 in PDE4C3). The focal point for the dimerization interface encompassing the ion pair has been defined in the core catalytic domain crystal structures and lies at the junction of helices-9 and-10 (Fig. 5a). Significantly, the tertiary structure of this region, with tightly folded short helices and loops, is expected to be dependent on its context within an intact core catalytic domain. Loss of the critical fold to this region in the short 25-mer sequences used in our peptide array assays might thus account for their failure to detect the dimerization surface encompassing Asp463. Arg499 (in contrast to Asp463) lies on a long helix (helix-11) and, while preservation of the helical secondary structure for this region might be reasonable in one or more of the array peptides, the residue is the sole point of dimer contact on helix-11 and lies 30 residues C-terminal to the focal residue set for the dimer interface (460-MYNDSSVLEN-469 … R499). Within the context of our peptide array, therefore, it is not surprising that peptides encompassing Arg499 as the single point of contact would fail to bind the PDE4D5 protein probe. Despite this, the Arg499–Asp463 ion pairing clearly makes an essential contribution to dimerization because the single charge reversal mutation of either one of this pair of amino acids serves to ablate the functional integrity of the catalytic unit dimer surface (Fig. 5c and see [50]). The acute sensitivity of the interface to the *trans* interaction of these two charged residues is further highlighted by the ability of a second and complementary Asp463Arg charge reversal mutation to rescue the dimerization capacity that is compromised by Arg499Asp alone (Fig. 5c).

**Fig. 9:**
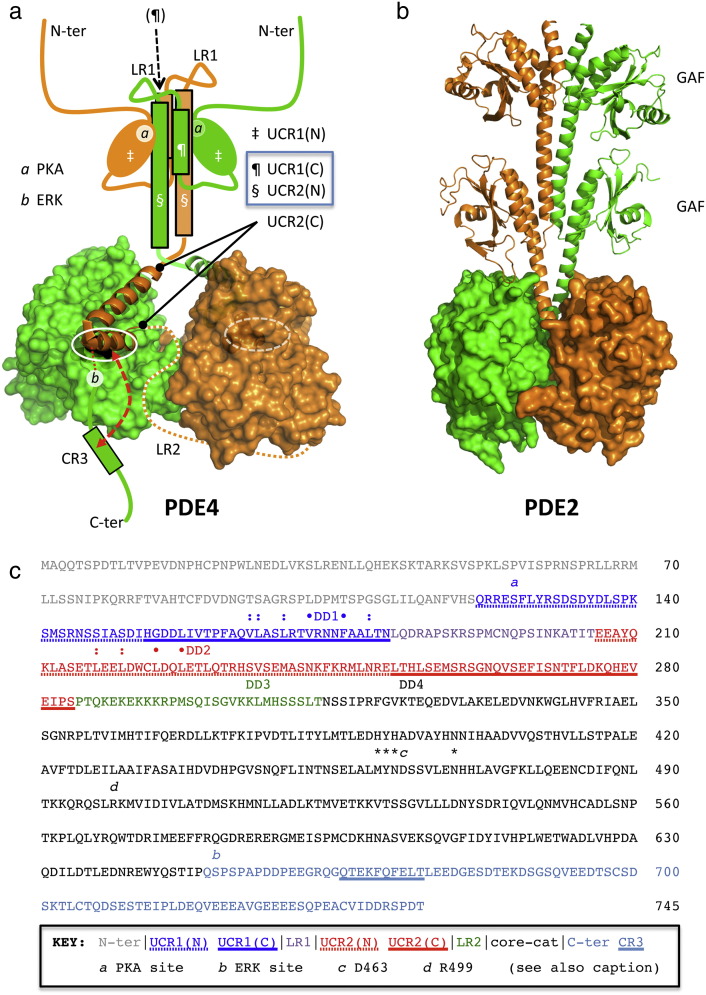
PDE4 long form dimerization model and sequence key. (a) Cross-capped model for PDE4 long form assembly based on the 3G45 UCR2-capped core catalytic domain crystal structure of Burgin et al. [68]. Sequence analysis fitted to a helical wheel model suggests that the UCR1(C) and UCR2(N) regions likely adopt helical 2° structure with extensive hydrophobic contact surfaces suitable for helix bundle assembly [37]. Inter-helix structural transition [*e.g.* mediated by phosphorylation (PKA site *a*; [23–26])] is proposed to control stability of the UCR2(N) cap and thence gate substrate entry to the catalytic pocket (white oval). The stability of the UCR2 cap is also affected by additional phosphorylation (ERK site *b*; [28,29,70,71]) and competitive capping with a C-terminal regulatory sequence (CR3; [68]) may further influence enzyme activity. The definition of LR2 (Linker Region 2) was coined [17] as that region of amino acids that joins UCR2 to the catalytic unit and is unique to each of the four PDE4 sub-families. (b) Structure of the PDE2 dimer (PDB: 3IBJ, [40]; see Discussion). (c) PDE4D5 sequence key, highlighting domain boundaries [15,47] and residues implicated in PDE4 long form dimer assembly. Residues marked: and • are proposed to contribute to extended hydrophobic contact surfaces along helical 2° structure motifs for UCR1(C) and UCR2(N) [42]. Quadruple alanine mutagenesis of the VFLL set (•) reportedly ablates PDE4D3 dimerization [42] and in the present work also compromises PDE4D5 dimerization. Sequences identified as possible dimerization domains in the peptide array studies of the present work are identified (DD1, 173-RNN-175; DD2, 228-ETL-230; DD3, 306-LMH-308; DD4, 323-KTE-325). PKA and ERK phosphorylation sites are marked (*a* and *b* respectively). Focal residues contributing to the core catalytic domain dimerization interface are marked (*, *c*, *d*), where *c* and *d* are respectively D463 and R499 and form an important ion pairing that underpins the stability of this interface (*cf.*Fig. 5a).

Although PDE4 core catalytic domain constructs that have been engineered to remove UCR1 and UCR2 can be crystallised in a dimeric state, such proteins do not exist as dimers in solution. Similarly, physiological PDE4 short forms that lack UCR1 fail to dimerize to a detectable extent [41–43]. Interestingly, however, evidence of a small dimer population, involving the PDE4D core catalytic unit was gained using glutaraldehyde cross-linking [50]. While this supports the existence of a monomer–dimer equilibrium involving the catalytic unit, even if it is heavily biased towards the monomer, irreversible glutaraldehyde cross-linking will inevitably perturb such equilibrium in favour of dimer detection by creating a pool of trapped dimers that are removed from equilibration with the monomer pool. This suggests that the free energy derived from engagement of the interface on the core catalytic domain is, on its own, insufficient to drive a monomer–dimer equilibrium in favour of the dimer; it takes the added presence of the UCR1 module, found only in PDE4 long forms, to favour dimer assembly through the establishment of an extra dimerization interface. This twin interface assembly likely underpins key regulatory aspects of PDE4 long form enzymology that involve auto-inhibitory capping of the catalytic pocket by a helical motif within the C-terminal half of UCR2. Thus, Burgin et al. have acquired crystal structures for the PDE4 core catalytic domain linked to a fragment from UCR2(C) that strongly support the notion that substrate entry to the catalytic pocket is gated by UCR2 [68]. In the available structures to date the LR2 linker region (Linker Region 2; [17]) that connects UCR2 to the core catalytic domain is disordered; this, combined with engineering in the linker region needed to acquire suitable crystals, prevented unambiguous assignment of the UCR2 capping mode to one or other of the two possibilities, namely intra-molecular *versus* inter-molecular. Nevertheless, an inter-molecular interaction, which would reflect cross-capping within a dimeric assembly, has been proposed as an attractive possibility [69]. Part of the rationale for that suggestion was the observation that the two connection sites for the missing N-terminal sequences in the Burgin structures are perfectly poised for a dimeric assembly of the UCR2(N) and UCR1(C) helices inferred from the work of Richter and Conti [42]. The model thus implicated for PDE4 long form dimer assembly is illustrated in Fig. 9a and invokes a 4-helix bundle organisation to the UCR1(C)/UCR2(N).

Such a dimerization model exhibits striking parallels but also some key differences with the established dimer structure for PDE2 (Fig. 9b). PDE2 shares a similar core catalytic domain fold to PDE4, but the UCR regulatory modules are replaced by tandem GAF domains. Pandit and co-workers have shown that PDE2 assembles as a dimer through pairing of the GAF domains so that regulatory cGMP binding to these units drives structural transition into the core catalytic domain through a coiled-coil assembly [40]. In particular, the PDE2 catalytic activity is controlled by regulatory deformation of the H-loop into the catalytic pocket (Fig. 5a, blue arrows) driven by motion in the coiled-coil assembly (Fig. 9b). In contrast to PDE2, the regulatory H-loop region is adapted as a core catalytic domain dimerization interface in PDE4. Moreover, the helices connecting the PDE2 GAF units to the catalytic domain are replaced in PDE4 by the UCR2(C) helices and a flexible linker (LR2; [17]), so that substrate access is controlled by UCR2 capping rather than by H-loop deformation. Such an assembly would then provide a means of controlling PDE4 long form catalytic activity through PKA phosphorylation-dependent induction of inter-helix structural transition in the UCR1(C)/UCR2(N) helix bundle to alter the stability of the UCR2(C) cap on the catalytic pocket.

Absolute confirmation of the cross-capping interaction and elucidation of the structural detail for the proposed UCR1(C)/UCR2(N) helix bundle assembly will require more advanced structural studies. However, our results here combined with those of Richter and Conti [42] firmly implicate the 172-VRNNF-176 sequence within UCR1(C) and the 224-LDQLETL-230 sequence within UCR2 as focal contributory elements. A more extended set of hydrophobic residues proximal to these sequences has also been implicated by Richter and Conti [42], as summarised in Fig. 9c, and thus our DD1 triple-alanine mutation of 173-RNN-175 in UCR1 may weaken but not necessarily fully disengage the UCR interface under circumstances where the core catalytic domain surface is still fully competent. At present there is no direct evidence to support H-loop deformatory transitions at the core catalytic domain dimerization interface, but structural studies of long and short isoforms, rather than just catalytic units, will be needed in order to fully comprehend differences consequent on monomer–dimer inter-conversion. This will be challenging, given the already demonstrated requirement for considerable mutagenesis to stabilise the construct in the currently available UCR2-capped structures [68].

Our peptide array studies also identified a potential interaction surface within the PDE4D catalytic unit that we have called DD4 (Figs. 3c, 4a). While mutation studies showed that DD4 did not play a critical role in stabilising PDE4D5 dimer it is interesting that K323, which forms part of DD4, provides the site of modification by sumoylation in PDE4D5 [33]. It may be that such a modification could affect this interface in such a way as to have functional consequences relating to alterations in the dimerization state/structure of PDE4D5.

We show here using PDE4D5 as an exemplar that PDE4 long isoforms adopt a dimeric state in living cells. This modality is exclusive to long isoforms as it critically depends upon not only an interaction involving a site within the conserved PDE4 catalytic unit but also one within the UCR1 regulatory region unique to PDE4 long isoforms. In other words, both sites in long forms can each separately stabilise the dimeric configuration. As such this may provide a fail-safe system for stabilising the dimeric state in PDE4 long forms. However, they also offer opportunities for specific informational inputs to destabilise the dimeric status, but only if destabilising actions are directed at both of these sites, which would then lead to the generation of a structurally and functionally distinct monomeric species. Thus it remains to be discovered whether long form monomer–dimer equilibrium can be altered by the action of various kinases able to phosphorylate residues within UCR1 and the PDE4 catalytic unit [23–26,28–30,70,71]; through mono-ubiquitination by the β-arrestin-sequestered E3 ligase, Mdm3 [32]; as a consequence of mutation of residues in UCR1 of PDE4D as seen in acrodysostosis (*e.g.* Q164P and L166S in PDE4D5; [72]) and when interacting with various partner proteins that sequester PDE4 isoforms to underpin compartmentalised cAMP signalling [1]. Structural studies of long and short isoforms, rather than just catalytic units, are needed in order to comprehend differences consequent on monomer–dimer inter-conversion.

Thus, the behaviour of PDE4 long forms is likely to be inherently distinct from that of PDE4 short forms as a direct consequence of the difference in quaternary structure, and doubtless this will contribute to the distinct physiological roles fulfilled by different PDE4 isoforms.
